# Evaluating the Effects of a Topical Preparation with Dexpanthenol, Silbiol, Undecylenic Acid, and Lidocaine on Palatal Mucosa Wound Healing in a Rat Model

**DOI:** 10.4274/balkanmedj.galenos.2018.2018.0167

**Published:** 2019-02-28

**Authors:** Yasemin Sezgin, Mehtap Bilgin Çetin, Şule Bulut, Nilgün Özlem Alptekin, Pelin Börçek

**Affiliations:** 1 Department of Periodontology, Başkent University School of Dentistry, Ankara, Turkey; 2 Department of Pathology, Başkent University School of Medicine, Ankara, Turkey

**Keywords:** Palate, periodontal surgery, rats, topical drug, wound healing

## Abstract

**Background:**

Postoperative complications occur after periodontal plastic surgeries, but an ideal treatment to overcome them has not been found yet.

**Aims:**

To evaluate the effects of topically applied Oral-norm gel on the healing of excisional wounds.

**Study Design:**

Animal experiment.

**Methods:**

Excisional wounds with a diameter of 3 mm were made in the center of the palatal mucosa of 63 Sprague Dawley rats. Seven animals were sacrificed at time 0. The remaining rats were divided into two groups: a test group in which the topical Oral-norm gel was applied three times a day and a control group in which nothing was applied. Seven animals in each group were sacrificed at 3, 7, 14, and 21 days. Mean wound surface area was measured photographically, while wound healing and width were evaluated microscopically.

**Results:**

The mean wound surface area decreased significantly after 3 days in both groups (p<0.001). Between days 3 and 7, the mean wound surface area decreased from 6.62 (2.85) to 0.83 (1.62) mm^2^ in the control group and 5.07 (0.88) to 1.42 (1.67) mm^2^ in the test group. The wound width decreased significantly on day 7 in both groups (p<0.001), with no further changes by day 14. Both groups had a significant increase in inflammation and vascularization on day 3 (p<0.001), with a reduction thereafter. No significant differences in macroscopic and microscopic measurements were observed between the groups at any time point (p>0.05).

**Conclusion:**

The Oral-norm gel has no positive healing effects in the palatal mucosa of rats.

Periodontal plastic surgeons typically use the palatal masticatory mucosa as a donor source for free gingival grafts because of its anatomic advantages and ideal tissue thickness (1). However, postoperative complications such as persistent bleeding, pain, paresthesia, and discomfort have been reported following a free gingival graft procedure (2,3). Although different adjunct treatments such as antiseptics, antibacterials (4,5), hemostatic agents (6), and bioactive materials (7) have been tested to overcome these complications and minimize patient discomfort, an ideal treatment has not been found to date. An increased wound healing rate can reduce the potential for infection and discomfort after periodontal plastic surgeries (8).

Oral-norm gel (Riga LMP Ltd., Latvia) is a combination of dexpanthenol, silbiol, undecylenic acid, and lidocaine. Dexpanthenol is the stable alcohol form of pantothenic acid. Within tissues, dexpanthenol is oxidized to pantothenic acid and stimulates proliferation of fibroblasts and acceleration of re-epithelialization in wound healing. Dexpanthenol not only hydrates but also protects both the mucous membranes and the skin (9,10). In addition, pantothenic acid supports the cellular antioxidant system that has a significant role in cellular defense and the repair systems against oxidative stress and inflammatory response (11,12). Silbiol is the trade name of the biologically active complex obtained from spruce or pine needles that have both anti-inflammatory and analgesic effects (13). Undecylenic acid inhibits the morphogenesis of *Candida albicans* (14). Lidocaine is a well-known, safe, and effective local anesthetic. Topical application of 1% lidocaine cream to a traumatic wound or an aphthous ulcer produces a significant reduction in pain intensity (15).

The complications that have been reported after periodontal plastic surgeries can be reduced by rapid healing. Because of the anti-inflammatory, analgesic, antioxidant, and wound-healing properties of its active components, we hypothesized that the Oral-norm gel would enhance the healing of the palatal donor area and reduce the complications that have been reported following free gingival graft procedures. In this study, we aimed to evaluate the effects of topically applied Oral-norm gel on the healing of excisional wounds in the center of the palatal mucosa of rats.

## MATERIALS AND METHODS

In the present study, 63 female Sprague Dawley rats weighing an average of 250-280 g were used. The animals were obtained from the Department of Medical Science Application and Research Centre of Başkent University. These animals were kept at room temperature 22±1 °C with a 12-h light/12-h dark cycle and were fed with commercial rat chow and water ad libitum. Permission from the Governmental Animal Protection Committee was obtained, and the study followed the Helsinki Declaration. The Animal Ethics Committee of Başkent University approved all of the experimental procedures.

**Figure 1:**
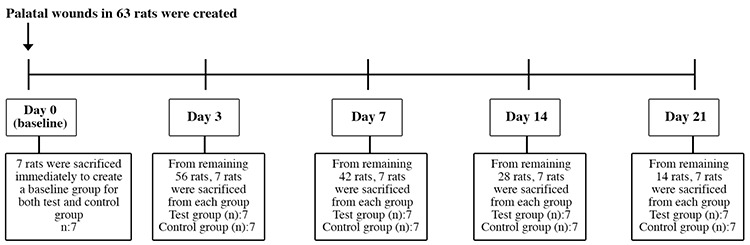
Timeline of the experiment.

The animals were anesthetized intraperitoneally with ketamine (50 mg/kg) and 2% xylazine (8 mg/kg). A disposable punch biopsy tool was used to create a circular 3 mm diameter full-thickness excisional wound in the center of the palatal mucosa (16). Mucoperiosteal specimens were removed with sharp dissection, exposing a circular area of uncovered bone to allow for secondary healing (17). Immediately after the excisional wound was made, seven animals were sacrificed, and they constituted the baseline group at time 0. This baseline group served as a common baseline group for both test and control groups. The remaining 56 animals were randomly divided into test and control groups. In the test group, the topical Oral-norm gel was applied three times a day. No adjuncts were used in the control group. In the test group, a syringe with a blunt cannula was used to apply 1 mL of Oral-norm gel to the wound. The animals were not given any food or drink until 2 h after application of the agent. Following the wound creation, seven animals in each group were sacrificed at 3, 7, 14, and 21 days using cardiac puncture under intraperitoneal anesthesia with ketamine hydrochloride (35 mg/kg) and xylazine (3 mg/kg; Figure 1). The animals were decapitated; their maxillae were separated, and the specimens were evaluated grossly and microscopically. The same investigator carried out all surgical operations under aseptic conditions.

### Macroscopic evaluation

A standard distance and magnification were used to photograph the palate specimens with Canon EOS 600D digital camera (Canon, Tokyo, Japan) (4). Standardization was achieved by using a tripod with the camera. A scientific ruler was also placed within the photographic area to confirm accuracy. The digital photographs were transferred to a computer for digital analysis. Following that, wound margins were marked to calculate mean wound surface area with an image processing program (Image J 1.34 s; US National Institutes of Health, Bethesda, MD, USA) using the ruler in the photograph as a scale reference. A blind-folded researcher coded the data to avoid observer bias.

### Microscopic evaluation

### *Histologic evaluation*

After digital imaging, the specimens were fixed for 24 h in 10% formalin. Following that, the specimens were decalcified in 10% formic acid for 2 weeks. The wound area of each specimen was sampled perpendicular to the midline of the palate. After routine tissue processing, the specimens were embedded in paraffin. For each wound, five serial sections, 5 μm apart, were cut perpendicular to the midline of the palate and stained with hematoxylin and eosin. Light microscopy was used to analyze the sections.

Inflammation and vascularization were scored as follows: 0= absent, 1= mild, 2= moderate, and 3= marked, according to Kirchner et al. (18). The density of the collagen fibers was scored using the following scale: 1= few collagen fibers, 2= few and partially spread collagen fibers, 3= few and fully spread collagen fibers, and 4= dense collagen fibers (19).

### *Histomorphometric evaluation*

Wound width was measured microscopically using a software system (cellSens Master Software V1.11, Shinjuku-ku, Tokyo, Japan) adapted for microscopic use by a blinded histologist.

### Statistical analysis

### *Sample size estimation*

The primary aim of this study was to compare the differences of wound surface area between the control and test groups. A sample size of seven cases for each group was required to detect at least 50% difference in wound surface area with an effect size of 2.29 between any of the two independent groups within a certain measurement time, in terms of the Bonferroni correction with a power of 85% at the 1.25% significance level. The difference of 50% was taken from literature (4). Sample size estimation was performed by using G*Power (Franz Faul, University of Kiel, Kiel, Germany) version 3.0.10.

Data analysis was performed using SPSS for Windows version 11.5 (SPSS Inc., Chicago, IL, USA). The Kolmogorov-Smirnov test was used to assess the continuous variables for a normal distribution. Levine’s test was used to evaluate the homogeneity of variances. The data are presented in the format median (interquartile range). The differences in the ranked means between groups were compared with the Mann-Whitney U test. The Kruskal-Wallis test was used to compare more than two independent groups. When the p value of the Kruskal-Wallis test was statistically significant, a Conover’s non-parametric multiple comparison test was performed to identify specific between-group differences. A p value less than 0.05 was considered statistically significant. For all possible multiple comparisons, a Bonferroni correction was applied to control for type I error.

## RESULTS

### Macroscopic evaluation

**Figure 2:**
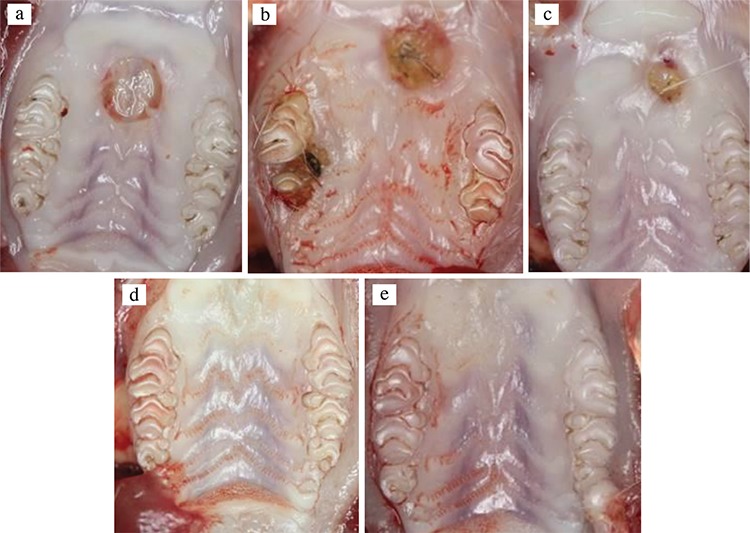
Clinical photographs of the palatal wounds showing gradual healing taken at days 0 (a), 3 (b), 7 (c), 14 (d), and 21 (e) from the test group.

**Figure 3:**
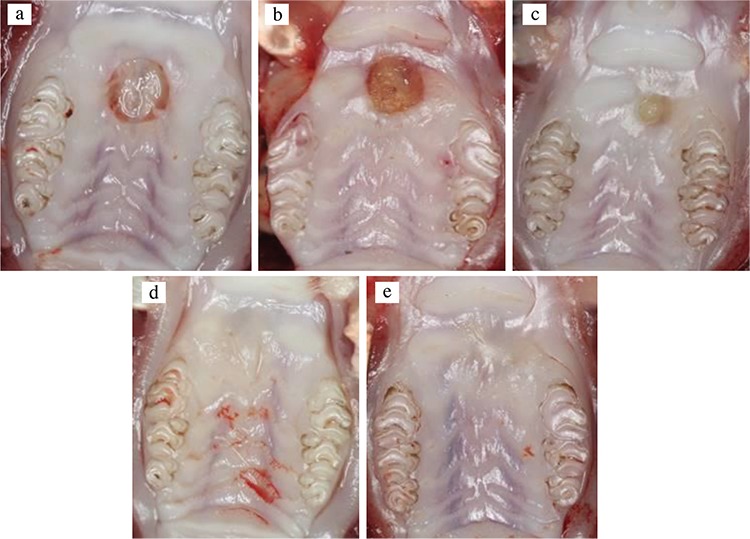
Clinical photographs of the palatal wounds showing gradual healing taken at days 0 (a), 3 (b), 7 (c), 14 (d), and 21 (e) from the control group.

The mean wound surface area measurements for the test and control groups at each time point are shown in Table 1. The wound surface covered 6.63 (0.75) mm^2^ immediately after the injury. In both groups, the mean wound surface area was unchanged on day 3 compared with baseline. However, the mean wound surface area was significantly decreased in both groups on days 7 and 14 (p<0.001). From days 14 to 21, no further reduction in mean wound surface area was observed in both the test and control groups (Table 1). Although changes in mean wound surface area were observed in both groups, there were no statistical differences among wound size between the test and control groups at any time period (p>0.05; Figures 2, 3).

### Microscopic evaluation

### *Histomorphometric examination*

**Figure 4:**
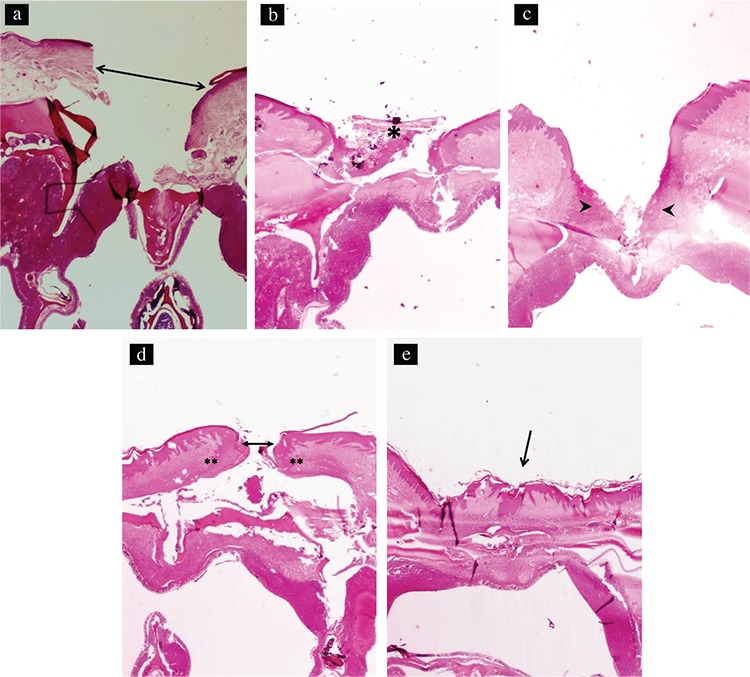
Photomicrograph of the palatine mucosa of the control group taken at day 0. The arrow shows the mucosal defect. No inflammation is observed (hematoxylin and eosin; original magnification x20) (a). Photomicrograph of the palatine mucosa of the control group taken at day 3. The asterisk shows the ulceration at the area of excision (hematoxylin and eosin; original magnification x20) (b). Photomicrograph of the palatine mucosa of the control group taken at day 7. The arrowheads show marked inflammation at the base of the ulcer (hematoxylin and eosin; original magnification x20) (c). Photomicrograph of the palatine mucosa of the control group taken at day 14. The edges of the mucosal defects are closer (arrow), and moderate inflammation at the base of the ulcer is noted (double asterisks; hematoxylin and eosin; original magnification x20) (d). Photomicrographs of the palatine mucosa of the control group taken at day 21. The arrow shows complete healing of the mucosal defect (hematoxylin and eosin; original magnification x20) (e).

**Figure 5:**
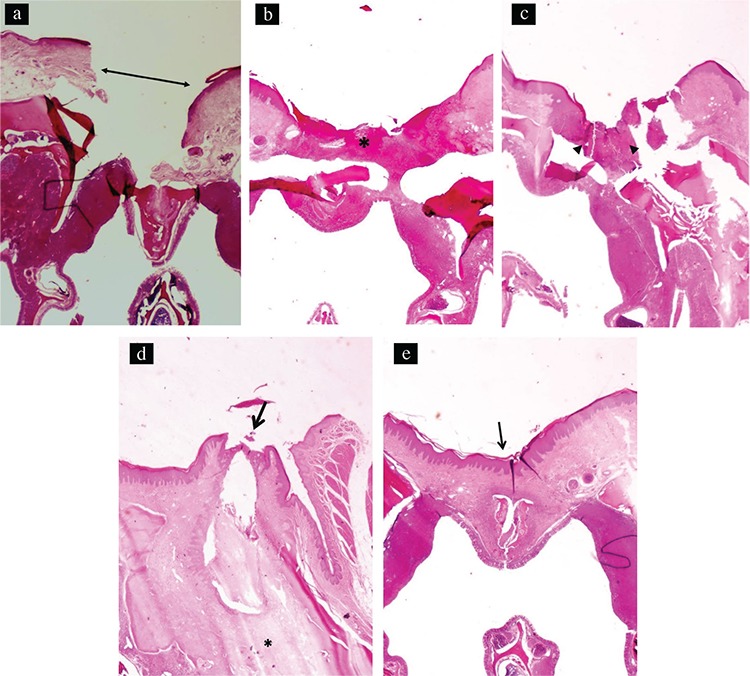
Photomicrograph of the palatine mucosa of the test group taken at day 0. The arrow shows the mucosal defect (hematoxylin and eosin; original magnification x20) (a). Photomicrograph of the palatine mucosa of the test group taken at day 3. The asterisk shows ulceration and prominent inflammation at the area of excision (hematoxylin and eosin; original magnification x20) (b). Photomicrograph of the palatine mucosa of the test group taken at day 7. The arrowheads show marked inflammation at the base of the ulcer obliterating the mucosal defect (hematoxylin and eosin; original magnification x20) (c). Photomicrograph of the palatine mucosa of the test group taken at day 14. The edges of mucosal defects almost emerge (arrow), and mild fibrosis at the base of the mucosa is noted (asterisk; hematoxylin and eosin; original magnification x20) (d). Photomicrographs of the palatine mucosa of the test group taken at day 21. The arrow shows complete healing of the mucosal defect (hematoxylin and eosin; original magnification x20) (e).

The distance between the wound margins of the test and control groups at different time points is shown in Table 2. Similar healing patterns were observed in both groups. The width of the wound did not change between days 0 and 3, but it decreased significantly by day 7. No further significant decrease was observed 7 days postoperatively (Figures 4, 5). No statistically significant difference was observed between the test and control groups at any time point during the experiment.

### *Histologic examination*

The inflammation scores for the test and control groups at each time point are shown in Table 3. On day 3, both groups showed a significant increase in inflammation compared with baseline (p<0.001). Both groups had a significant decrease in inflammation on day 7 compared with day 3 (Figures 4, 5). The inflammation scores continued to decrease significantly in the test group by day 14, and this remained unchanged on day 21 (Figure 5). In the control group, the reduction of inflammation on day 14 was not statistically significant compared with day 7. However, the reduction on day 21 was statistically significant compared with days 14 and 7 (Figure 4). Despite this difference, there were no significant differences in inflammation between the test and control groups at any time point during the experiment (p>0.05; Table 3).

On day 3, both groups showed significant increases in vascularization compared with baseline (p<0.001). In the test group, the vascularization reduced significantly on days 7 and 14 compared with day 3, and this remained unchanged on day 21. In the control group, a statistically significant reduction was observed on days 14 and 21 compared with days 3 and 7. There were no statistically significant differences between the groups at any time point during the experiment (p>0.05; Table 4).

The density of the collagen fibers did not increase on day 3 compared with baseline in both groups. In the test group, a statistically significant increase in collagen fiber density was observed on days 7, 14, and 21 compared with days 0 and 3. In the control group, a statistically significant increase in collagen fiber density was noted on days 14 and 21 compared with day 0. There were no statistically significant differences in collagen fiber density observed between the test and control groups at any time point during the experiment (p>0.05;Table 5).

## DISCUSSION

The present study was conducted to test the effects of the Oral-norm gel on excisional wound healing by secondary intention. Because of previously reported complications following free gingival grafts (2,3), different agents have been tested to reduce patient morbidity. However, no therapy has been devised to completely address these challenges. The results of this study showed that although excisional wounds created in the center of the palate of 63 Sprague Dawley rats healed uneventfully with the Oral-norm gel treatment, no positive healing benefit of the gel was observed.

The palatal excisional wound model utilized in the present study was previously used to investigate intraoral wound healing or factors that might affect it. This wound model creates a reproducible wound that could be followed clinically and histologically (4,5,17,20). In this study, we used a wound area calculation method that was previously introduced by Hammad et al. (5). This method standardizes digital photographs that have been magnified by a computer, allowing for the assignment of wound boundaries and a surface area calculation using Image J software. This reduces the influence of human error that might be present in other methods (5).

The histomorphometric evaluation technique used to calculate the wound width in our study was based on previously described techniques (5,17). One-dimensional analysis of the wound may be considered as a limitation for this evaluation technique. According to the macroscopic and microscopic results of the present study, the wound dimensions were reduced significantly over time. These findings are consistent with the results of previous studies in which similar wounds were created, and complete healing in most animals was observed 21 days after injury (17).

Wound healing is a complex process that includes three phases: an inflammatory phase, a proliferative phase, and a remodeling phase (21). Migration of platelets, neutrophils, macrophages, and lymphocytes to the wound occurs in the inflammatory phase. During the proliferative phase, the number of fibroblasts and macrophages increases. The final remodeling phase is characterized by extracellular matrix recreation and collagen deposition mediated by fibroblasts (16).

The main purpose of using the Oral-norm gel to promote wound healing in this study was based on its anti-inflammatory, analgesic, antioxidant, and wound-healing properties. Re-epithelialization is a major component of the wound-healing process and necessary for final wound closure. The topical application of dexpanthenol is widely used to stimulate skin regeneration and promote wound healing (10,22). It is also used to manage post-tonsillectomy pain and mucosal healing (23). Although rapid wound closure was expected because of the dexpanthenol (9) found in the Oral-norm gel, in the present study, no statistically significant differences were observed in the wound width or mean wound surface area.

Previous studies (13) have found that silbiol has anti-inflammatory and analgesic properties. These benefits are linked to the presence of polyphenolic compounds in the silbiol extract. Dexpanthenol also increases the levels of reduced glutathione and coenzyme A, which play a major role in cellular defense and in the repair of damage caused by oxidative stress and inflammation (12,24). Because of the anti-inflammatory, analgesic, and antioxidant properties of both of these ingredients, it was hypothesized that the Oral-norm gel could shorten the inflammatory phase of wound healing and therefore accelerate wound closure. However, in the present study, no statistically significant macroscopic or microscopic changes were noted between the Oral-norm-treated and control samples.

To the best of our knowledge, there are no prior studies that evaluated the effect of the Oral-norm gel on the healing of excisional wounds of the palate. A direct comparison with prior work is therefore not possible. Based on the present results, the Oral-norm gel did not enhance the healing of the excisional wounds that were created in the palate of the rats. The application method of the agent used in the present study is similar to techniques successfully used in the past (4,5). However, similar healing patterns may have been observed in both groups because of the insufficient protection of the agent against oral fluids or small sample size. The inability of our model to offer sufficient protection of the Oral-norm gel from oral fluids is one limitation of our study. We may, therefore, consider the immediate placement of an acrylic stent to stabilize the material in future human studies. Another limitation of this study is the lack of an immunohistochemical analysis of growth factors and cytokines. After tissue damage, a series of cellular and molecular processes are set in motion with the concomitant involvement of cytokines, growth factors, and proteases to complete wound repair (25,26). Cytokines and growth factors are therefore useful biomarkers of wound healing. By quantifying the growth factors or cytokines that are critical to the wound-healing process, a more accurate evaluation of the Oral-norm gel may be possible. Studies with a larger number of samples and immunohistochemical analysis of changes in the growth factor or cytokine levels are required to confirm these findings.

## Figures and Tables

**Table 1:**
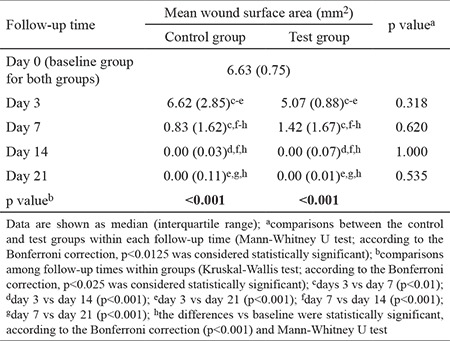
Mean wound surface area evaluated at five time points

**Table 2:**
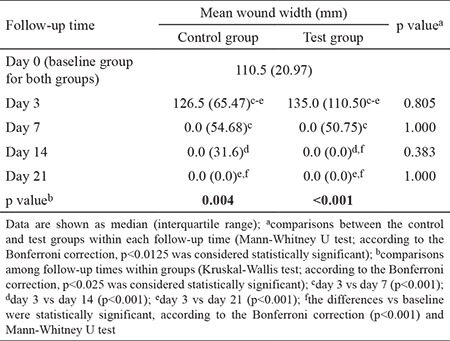
Wound width measurements evaluated at five time points

**Table 3:**
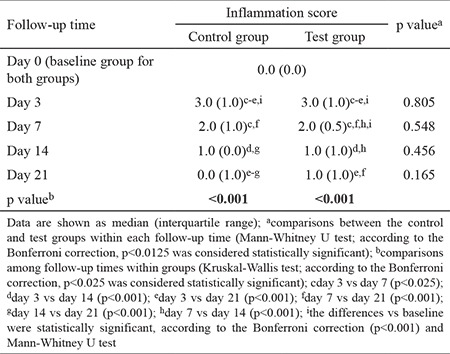
Inflammation scores evaluated at five time points

**Table 4:**
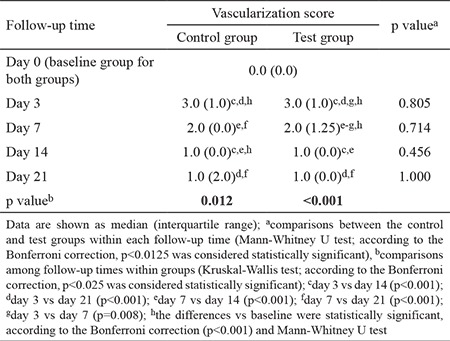
Vascularization scores evaluated at five time points

**Table 5:**
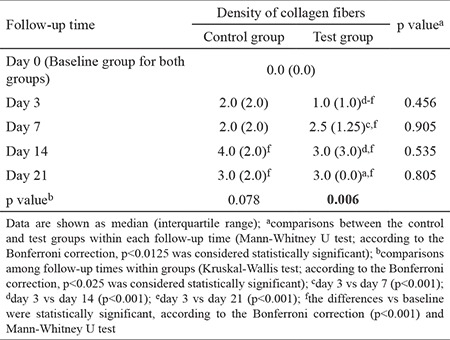
Density of collagen fibers evaluated at five time points
